# Cold Ethanol Extraction of Cannabinoids and Terpenes from Cannabis Using Response Surface Methodology: Optimization and Comparative Study

**DOI:** 10.3390/molecules27248780

**Published:** 2022-12-11

**Authors:** Philip Wiredu Addo, Sai Uday Kumar Reddy Sagili, Samuel Eichhorn Bilodeau, Frederick-Alexandre Gladu-Gallant, Douglas A. MacKenzie, Jennifer Bates, Garnet McRae, Sarah MacPherson, Maxime Paris, Vijaya Raghavan, Valérie Orsat, Mark Lefsrud

**Affiliations:** 1 Bioresource Engineering Department, Macdonald Campus, McGill University, Ste-Anne-De-Bellevue, Montreal, QC H9X 3V9, Canada; 2 EXKA Inc., 7625 Route Arthur Sauvé, Mirabel, QC J7N 2R6, Canada; 3 National Research Council of Canada, Metrology, 1200 Montreal Road, Ottawa, ON K1A 0R6, Canada

**Keywords:** cannabis, cannabinoids, cold ethanol, delta-9-tetrahydrocannabinol, extraction

## Abstract

Efficient cannabis biomass extraction can increase yield while reducing costs and minimizing waste. Cold ethanol extraction was evaluated to maximize yield and concentrations of cannabinoids and terpenes at different temperatures. Central composite rotatable design was used to optimize two independent factors: sample-to-solvent ratio (1:2.9 to 1:17.1) and extraction time (5.7 min–34.1 min). With response surface methodology, predicted optimal conditions at different extraction temperatures were a cannabis-to-ethanol ratio of 1:15 and a 10 min extraction time. With these conditions, yields (g 100 g dry matter^−1^) were 18.2, 19.7, and 18.5 for −20 °C, −40 °C and room temperature, respectively. Compared to the reference ground sample, tetrahydrocannabinolic acid changed from 17.9 (g 100 g dry matter^−1^) to 15, 17.5, and 18.3 with an extraction efficiency of 83.6%, 97.7%, 102.1% for −20 °C, −40 °C, and room temperature, respectively. Terpene content decreased by 54.1% and 32.2% for extraction at −20 °C and room temperature, respectively, compared to extraction at −40 °C. Principal component analysis showed that principal component 1 and principal component 2 account for 88% and 7.31% of total variance, respectively, although no significant differences in cold ethanol extraction at different temperatures were observed.

## 1. Introduction

Plant metabolites may be classified as primary or secondary based on their involvement in plant development and growth [1]. Although secondary metabolites are not directly involved in development and growth, they protect plants against biotic (insects, viruses, and bacteria) and abiotic stress (unfavourable environmental conditions) [1,2], add colour and odour, as well as attracting insects for pollination [3]. They are divided into three chemically distinct groups based on their synthesis: phenolics, terpenes, and nitrogen-containing compounds [4].

Major active secondary compounds in the cannabis (*Cannabis sativa*) plant include the terpenophenolic phytocannabinoids, a group of chemical compounds that alter neurotransmission activity in the brain by acting on cannabinoid receptors [5,6,7,8]. Phytocannabinoids were considered exclusive to cannabis for many years, until they were discovered in some liverwort and fungi species [9]. Luo et al. (2019) genetically modified brewer’s yeast (*Saccharomyces cerevisiae*) to produce two of the most common cannabinoids, tetrahydrocannabinol (THC) and cannabidiol (CBD). 

Extraction of secondary metabolites from plant biomass is the first step for any medicinal plant study [10,11,12,13]. Understanding the genetic composition, plant metabolite biosynthesis, and the prevention of metabolite degradation during postharvest, formulation of cannabis oil, and consumer consumption are important when selecting an extraction technique [14,15,16,17]. Commonly used extraction methods in the medicinal industry include the mechanical press and conventional Soxhlet systems. Low extraction rate and poor oil quality are the major disadvantages of the mechanical press system [18]. The Soxhlet extractor dates to 1879 and is based on the principle that the desired compounds are highly soluble in the solvent used, while impurities are insoluble [19,20,21]. Soxhlet extraction is normally performed at the boiling point of the solvent for an extended period, which can lead to thermal decomposition of the metabolites.

Addo et al. (2021) and Ubeed et al. (2022) reviewed studies on the cannabis industry showed that modern extraction techniques such as cold ethanol, supercritical CO_2_ extraction, ultrasound extraction, and microwave systems have been developed for medicinal plants to improve extraction efficiency and extract quality. Compared to supercritical CO_2_ extraction and other traditional extraction systems, cold ethanol extraction limits the extraction of chlorophylls and waxes; hence, it does not require an extract purification or winterization step [22,23,24]. Food-grade ethanol is commonly used as an extraction solvent as it is considered a “green” and GRAS (generally recognized as safe) solvent, although other solvents such as hexane and butanol reportedly improve extraction yields [25]. This study aimed to optimize cold ethanol for cannabis biomass extraction. Effects of independent variables, including sample-to-solvent ratios, extraction temperatures, and extraction times, on the crude oil yield and concentration of cannabinoids and terpenes were investigated. Response surface methodology was used to optimize the conditions and compare the effects of the dependent variables using quantitative results.

## 2. Results

### 2.1. Preliminary Cold Ethanol Extraction Results

The selection of independent variables and their ranges for the extraction systems were based on preliminary experiments and a literature review of the probable effects of the parameters on the yield of cannabis oil, cannabinoids, and terpenes (dependent variables). Cannabinoid and terpene chromatographs and concentrations for the biomass used in this work and a parallel study [26] were measured using the liquid chromatography-tandem mass spectrometer (LC-MS/MS) and gas chromatography-tandem mass spectrometer (GC-MS/MS), respectively. The cryo-ground biomass used for the study contained 17.9 g 100 g dry matter^−1^ (THCA), 0.17 g 100 g dry matter^−1^ (THC), and 0.04 g 100 g dry matter^−1^ (CBDA) as described previously [26]. The cannabis biomass used for this study can be classified as a Type I chemovar, according to the classification set by Lewis et al. (2018) based on the high concentration of THCA compared to CBDA. The total chromatographic run time was 18 min for the cannabinoids and 25 min for the terpenes. THCVA, which is produced from cannabigerovarinic acid (CBGVA), was 1.01 g 100 g dry matter^−1^ in the cryo-ground sample [26]. CBGVA is produced by the prenylation of divarinolic acid, instead of olivetolic acid, with geranyl diphosphate from terpenoid synthesis [27,28]. In contrast to THC, Δ9-tetrahydrocannabivarin (THCV), the decarboxylated form of THCVA, does not cause psychoactive effects and may be a useful metabolite for regulating weight loss and obesity as it decreases appetite and increases satiety and energy metabolism [29,30].

Cannabinoid concentration data acquired after cold ethanol extraction at −20 °C, −40 °C, and room temperature are summarized in Table 1. CBD, CBDA, total CBD, and other cannabinoids were not presented as their concentrations were below the limit of detection of the instrumentation and methodology. An observed increase in acidic cannabinoid concentrations indicates that cold ethanol extraction does not cause cannabinoid decarboxylation. This can be explained by the low temperature maintained during the extraction process. The statistical significance of the experimental factors on the cold ethanol extraction process, specifically cannabinoid, terpenes, and extraction yield, for each response, and linear, quadratic, and interaction coefficients of experimental factors are presented in Table 2. Regression intercepts of the developed models demonstrate significant (*p* < 0.05) relationships between the cold ethanol extraction independent variables at the set extraction temperatures and corresponding responses of the produced extracts. A positive regression coefficient indicates a positive correlation between the independent variable and the response.

### 2.2. Effects of the Extraction Factors on Experimental Responses

#### 2.2.1. Effect of Sample (g)-to-Solvent (g) Ratio

Central composite rotatable design (CCRD) is highly efficient in providing useful information on the effects of process parameters for optimization purposes with a reduced number of total experimental runs compared to factorial designs [31,32]. Sample-to-solvent ratio was the most important factor affecting cold ethanol extraction of cannabis, demonstrating a significant (*p* < 0.05) effect on extraction of cannabinoids, terpenes, and extraction yields for all experimental conditions. Decreasing the sample (g)-to-solvent (g) ratio significantly (*p* < 0.05) increased the extraction yield and concentration of cannabinoids and terpenes, likely by providing an increased surface area for the extraction of secondary metabolites from the trichomes. This is evident by the positive sample-to-solvent ratio coefficient values (β_1_). Krishnaswamy et al. (2013) showed that decreasing the mass of grape seeds (*Vitis vinifera*) in ethanol during microwave-assisted extraction, increased the total extracted phenols by 7%. Similar findings have been reported for microwave-assisted extraction of antioxidants from olive (*Elaeagnus angustifolia*) [16].

**Figure 1:**
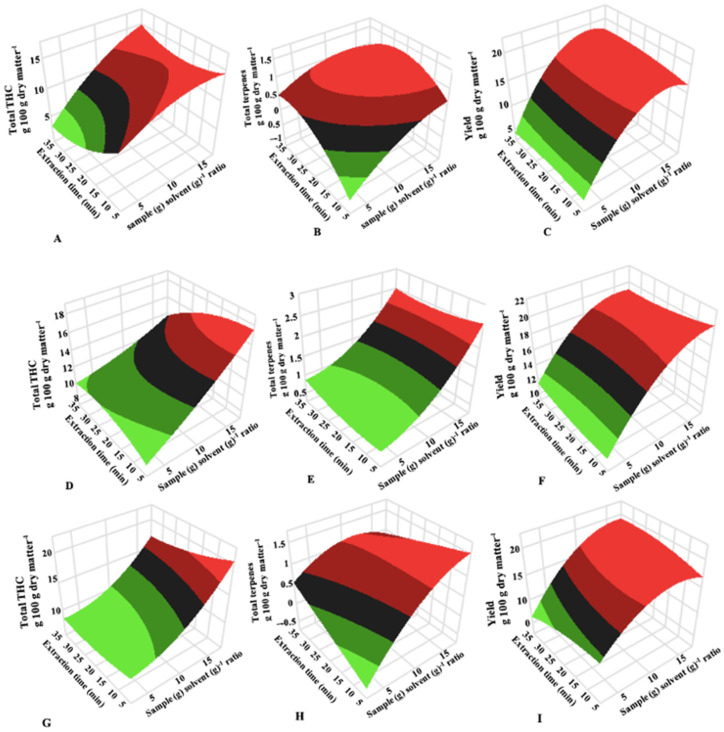
3D plots showing the combined effects of sample (g)-to-solvent (g) ratio and extraction time (min) on the concentration (g 100 g dry matter^−1^) of total THC (**A**,**D**,**G**), total terpenes (**B**,**E**,**H**), extraction yield (**C**,**F**,**I**) for cold ethanol extraction of cannabis at −20 °C (**A**–**C**), −40 °C (**D**,**E**), and room temperature (**G**–**H**).

Table 2 and Figure 1 showed that sample-to-solvent ratio had a significant (*p* < 0.05) interaction effect with extraction time only for extraction yield for cold ethanol extraction at room temperature. Response surface plots (Figure 1) showed that the extraction yield increased by 37.7%, 19.2%, and 23.8% when the sample mass in 40 mL of ethanol was reduced from 6.31 g to 2.1 g using 10 min extraction time for cold ethanol extraction at −20 °C, −40 °C, and room temperature, respectively. Similar observations were observed for the extraction time of 30 min used for this study.

Quadratic effect, β_11_ (sample-to-solvent^2^), significantly (*p* < 0.05) impacted extraction yield for cold ethanol extraction of cannabis at −20 °C (Table 2). For cold extraction at room temperature, the quadratic effect, β_11_, had significant (*p* < 0.05) effects on the THCA, total THC, CBG, CBGA, total CBG, THCVA, CBCA, and extraction yield. Importantly, data showed that improved extraction of cannabinoids and cannabis oil can be achieved with all three cold ethanol extraction systems used for the study. Significant difference (*p* < 0.05) was not observed for the extraction yield using either cold ethanol extraction at −20 °C, −40 °C, and room temperature. However, the extracted cannabis oil extracted at room temperature must be winterized to remove residual waxes and other heavier compounds. If a lower sample-to-solvent ratio is optimal, cost analyses of scale-up studies and industrial systems must be done to minimize the high cost of ethanol needed to maximize extraction yield and cannabinoid concentrations. Studies on ethanol recovery from residual biomass after extraction using either a mechanical press, centrifugal system, or vacuum filtration must be conducted.

#### 2.2.2. Effect of Extraction Time

Effect of extraction time for different plant biomass has been reported and the longer the extraction time, the higher the total amount of metabolites extracted according to mass transfer principles [17,33,34]. Some researchers have reported that extraction time can be reduced by increasing extraction temperature [34,35,36]. However, metabolite stability can decrease when they are exposed to high temperatures because most phytochemicals are sensitive to heat [24]. Szalata et al. (2022) showed compared to cold water extraction, hot water extraction significantly (*p* < 0.05) increased the CBD content from 0.01 to 0.06 g 100 g dry matter^−1^ and 0.01 to 0.05 g 100 g dry matter^−1^ in Futura 75 and KC Dora cannabis accessions, respectively. The increase in CBD can be attributed to the decarboxylation of CBDA to CBD due to the increase in extraction temperature. Optimization of extraction time and temperature to improve extraction yield must be based on the desired phytochemicals’ stability during extraction and energy cost analyses. Data presented in Table 2 and Figure 1 indicate that extraction time did not have a significant effect (*p* < 0.05) on the experimental responses during cold ethanol extraction of cannabis at −40 °C.

For cold ethanol extraction at −20 °C, extraction time significantly (*p* < 0.05) increased the total terpenes extracted from cannabis. Using the same sample-to-solvent ratio, extending the extraction time increased the concentration of total terpenes in the extracted cannabis oil, likely by increasing the contact time of the sample in the solvent. The negative coefficient values showed a negative significant (*p* < 0.05) correlation between extraction time and the concentration of THCA, total THC, CBGA, total CBG, THCVA, CBCA, and extraction yield for cannabis oil when performing cold ethanol extraction at room temperature (Table 2). This can be attributed to degradation or isomerization, which can affect analytical quantification [34,36]. Spigno et al. (2007) observed that the concentration of secondary metabolites, anthocyanin, and tannin, in grape (*Vitis vinifera*) diminished beyond 20 h extraction time. Quadratic effects, β_22_ (extraction time^2^), were only observed for total terpenes and extraction yield with cold ethanol extraction at −20 °C and cold ethanol extraction at room temperature, respectively.

### 2.3. Optimal Cold Ethanol Extraction Conditions for Cannabis

Based on the observed effects of the independent parameters used for the study, optimal cold ethanol extraction conditions for cannabis at different temperatures and the predicted responses at 95% confidence interval are listed in Table 3. Optimization was driven by maximum desirability and yield of cannabinoids, terpenes, and extracted cannabis oil. The desirability function consolidates all the responses into one response with a numerical value varying from 0 (one or more product characteristics are unacceptable) to 1 (all product characteristics on target).

Based on the data, cold ethanol extraction at −20 °C, −40 °C, and room temperature using a sample-to-solvent of 1:15 for 10 min are presented as the optimal conditions for maximum responses. According to these statistical analyses of the predicted responses, there were no significant (*p* < 0.05) differences between the extraction yields for the cold ethanol extraction performed at different temperatures. However, reducing the temperature of the cold ethanol extraction system from −20 °C to −40 °C slightly increased cannabinoid concentration by 7.8%. Compared to room temperature, cold ethanol extraction at −40 °C slightly increased the extraction yield by 6%. If a high terpene content is desired, cold ethanol extraction at −40 °C is recommended.

Concentration of extracted total terpenes was reduced by 54.1% and 32.2% for extraction at −20 °C and room temperature, respectively, compared to extraction at −40 °C. Cannabinoid concentrations in extracts were not significantly (*p* < 0.05) different between room temperature extraction and extraction at −40 °C. Compared to the reference ground sample [26], THCA concentration changed from 17.9 (g 100 g dry matter^−1^) to 15, 17.5, and 18.3 with an extraction efficiency of 83.6%, 97.7%, 102.1% for −20 °C, −40 °C, and room temperature, respectively. Extraction efficiency was calculated based on the concentration of THCA in extracts compared to the concentration of THCA in the reference cryo-ground biomass used for the study. Extraction efficiency greater than 100% for cold ethanol extraction at room temperature can be explained by the biosynthesis or the conversion of other cannabinoids to THCA during the extraction process or variance due to the analytical method [13]. Preliminary studies conducted showed that postharvest processing of cannabis can influence the biosynthesis of cannabinoids. The results showed a significant (*p* < 0.05) increase in the total THC (24.2 g 100 g dry matter^−1^) and THCA (27.2 g 100 g dry matter^−1^) concentrations in pre-frozen, undried samples compared to fresh, undried samples. Further studies evaluating the effect of cold temperature on biosynthesis of secondary metabolites, cannabinoids, and terpenes, at the molecular level must be conducted to explain the differences observed in this study.

### 2.4. Model Fitting

JMP software (JMP 4.3 SAS Institute Inc., Cary, NC, USA) was used for the least square multiple regression analysis of the data and model building. Summary of fit for the experimental data to each model is presented in Table 4. Results show non-significant (*p* > 0.05) lack-of-fit values for model A (full model), except for extracted oil and total terpenes for both extraction at −20 °C and −40 °C. Using model B, which excludes extraction time, all interaction and quadratic terms that include extraction time, only showed non-significant (*p* > 0.05) lack-of-fit values for total terpenes extracted at −20 °C and −40 °C. This indicates that there is a satisfactory level of accuracy of model B for explaining the relationship between the total terpene content in extracted cannabis using cold ethanol at either −20 °C or −40 °C and prediction of the corresponding responses. However, both proposed models do not adequately explain the extracted cannabis oil yield using cold ethanol extraction at −20 °C and −40 °C, and other extraction parameters must be considered to improve the extraction models.

Significant (*p* < 0.05) ANOVA *p*-values indicated significant differences between the extraction conditions. Coefficients of determination (R^2^) and adjusted R^2^ values of the developed model A ranged from 0.55 to 0.99 and 0.22 to 0.98. Higher R^2^ and adjusted R^2^ values imply that the experimental data successfully fit the equation with a low deviation from mean values. However, model A should be used when predicting responses for ethanol extraction at room temperature and model B for cold ethanol extraction at −20 °C and −40 °C.

### 2.5. Principal Component Analysis

**Figure 2:**
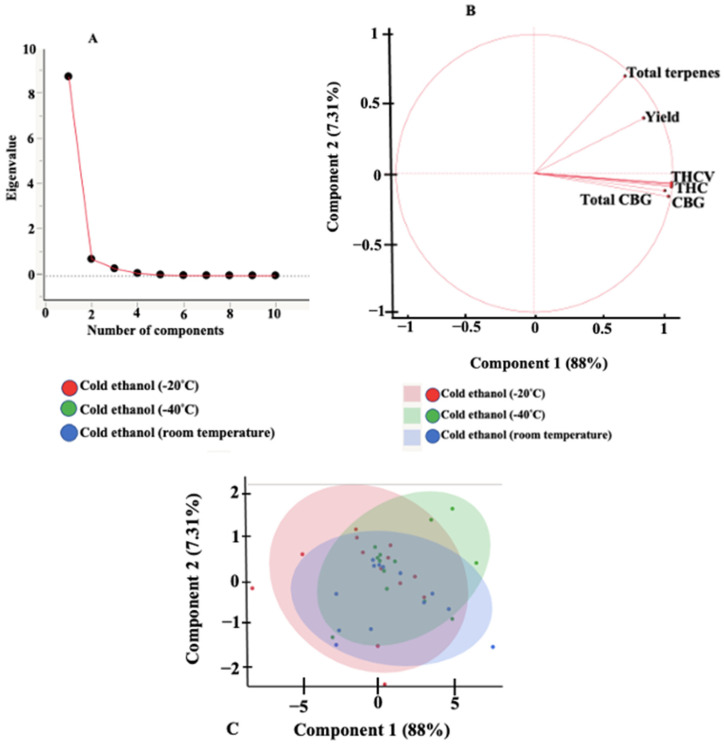
Principal component analysis plots for cold ethanol extraction of cannabis at various temperatures: −20 °C, −40 °C, and room temperature. Scree plot (**A**), loadings plot (**B**), and scatterplot (**C**).

An exploratory principal component analysis (PCA) was performed to help identify correlation and dependencies between the two independent variables, cannabis biomass sample-to-solvent ratio and extraction time. The scree plot, loadings plot, scores plot, and scatterplot for the different extraction systems are presented in Figure 2. A scree plot (Figure 2A) is a line plot of the eigenvalues of principal components and is used to determine the number of principal components that are responsible for variations in the data during PCA [37]. The scree plot indicated that the first two principal components (PC1 and PC2) account for 95.3% of the total variance (PC1 = 88% and PC2 = 7.3%). The loading plot (Figure 2B) provides information on how the responses contribute to the variations accounted for by the principal components [38]. The axes on the loading plot range from 1 to −1. The closer the value of the response on the graph to either −1 or 1 describes how strongly the response influences the component. A positive value on the loading plot indicates a positive correlation between the response and the PC. According to the loading plots, parameters positioned close to each other indicate a high positive correlation between them. An increase in the THCA content of an extract can be an indicator of an increase in THCVA. The major cannabinoids identified in the extracts are important contributors to PC1. The loading plot showed that total CBG, CBG, THCV, and THC account for most of the variation of PC1 and not for PC2. PC2 and PC1 can be explained by the total terpenes and the yield of extracts. Scatter plots (Figure 2C) did not show any variation in cold ethanol extraction at different temperatures (−20 °C, −40 °C, and room temperature). This is evident by the overlap of responses for cold ethanol extraction at different temperatures.

### 2.6. Verification of Models

Generated models for cold ethanol extraction of cannabis at various temperatures (−20 °C, −40 °C, and room temperature) were verified by conducting an extraction process using the optimal conditions, sample-to-solvent ratio of 1:15 for 10 min. The corresponding experimental values for cannabinoid content, total terpenes, and extraction yields were determined and compared to predicted results. Data show a strong correlation ranging from 0.87 to 0.93 between the predicted and experimental values, which indicates suitability of the models in predicting cannabinoid/terpenes profiles and extract yield for cannabis for optimum cold ethanol extraction at −20 °C, −40 °C, and room temperature.

## 3. Materials and Methods

### 3.1. Sample Preparation

Harvested cannabis inflorescences from three cannabis accessions, Qrazy Train, Qrazy Apple, and Qrazy Angel, cultivated indoors using the same growing conditions were obtained from EXKA Inc. (Mirabel, QC, Canada). Harvested inflorescences were pre-frozen at −20 °C for 24 h before transferring to a laboratory-scale vacuum freeze-dryer (Martin Christ Gefriertrocknungsanlagen GmbH Gamma 1−16 LSCplus, Osterode, Lower Saxony, Germany) with a condenser temperature of −55 °C. Freeze-drying was carried out at plate temperatures of 10 °C for 24 h at 0.85 mbar. The initial moisture content of the inflorescence ranged from 78.52% (wb) to 80.48% (wb). Freeze-dried inflorescence from the different cannabis accessions were mixed and cryo-ground to uniform particle size (0.25–0.5 mm) using liquid nitrogen and a mortar and pestle. Ground samples were kept in zip-locked plastic bags, manually homogenized, then stored at either −20 °C, −40 °C, or room temperature before extraction and analysis.

### 3.2. Reagents

Food-grade ethanol was purchased from Commercial Alcohols (Brampton, Ontario, Canada). Reference standards of cannabinoids and isotopically labeled cannabinoids were purchased from Cerilliant (Round Rock, TX, USA). All neutral cannabinoids including Δ9-THC (tetrahydrocannabinol), Δ8-THC, CBD (cannabidiol), CBG (cannabigerol), CBN (cannabinol), CBC (cannabichromene), THCV (tetrahydrocannabivarin), CBDV (cannabidivarin), CBGV (cannabigerivarin), and CBV (cannabivarin) were provided at 1.0 mg mL^−1^ in methanol. CBL (cannabicyclol) was provided at 1.0 mg mL^−1^ in acetonitrile. The acidic cannabinoids, including Δ9-THCA (tetrahydrocannabinolic acid), CBDA (cannabidiolic acid), CBGA (cannabigerolic acid), CBNA (cannabinolic acid), CBCA (cannabichromenic acid), THCVA (tetrahydrocannabivarin acid), CBDVA (cannabidivarinic acid), and CBGVA (cannabigerovarinic acid), were provided at 1.0 mg mL^−1^ in acetonitrile. CBLA (cannabicyclolic acid) was provided at 0.5 mg mL^−1^ in acetonitrile.

Isotopically labeled cannabinoids, including Δ9-THC-d_3_, CBD-d_3_, CBN-d_3_, and CBG-d_3_, were provided at 0.1 mg mL^−1^ in methanol while Δ9-THCA-d_3_, CBGA-d_3_, and CBCA-d_3_ were provided at 0.1 mg mL^−1^ in acetonitrile. THC-d_3_ was used as internal standard for Δ9-THC, Δ8-THC, THCV, CBC, and CBL. THCA-d_3_ was used for THCA, CBNA, and THCVA. CBD-d_3_ was used for CBD, CBDA, CBDV, and CBDVA. CBN-d_3_ was used for CBN and CBV. CBG-d_3_ was used for CBG and CBGV. CBGA-d_3_ was used for CBGA and CBGVA and CBCA-d_3_ was used for CBCA and CBLA. Ultrapure water was collected from a Millipore Milli-Q Advantage A10 mixed bed ion exchange system fed with reverse osmosis domestic water (Jaffrey, NH, USA). Optima^®^ grade acetonitrile, methanol, and formic acid were procured from Fisher Scientific (Fair Lawn, NJ, USA). 

Terpene reference standards were purchased from Restek (Bellefonte, PA, USA) and provided at 2.5 mg mL^−1^ in isopropanol. Isotopically labeled terpene (±)-linalool-d3 (vinyl-d3) was purchased from CDN Isotopes (Pointe-Claire, Quebec, Canada) and used as an internal standard. Hexane (HPLC Plus, ≥95%) was purchased from Millipore-Sigma (Oakville, ON, Canada).

### 3.3. Cold Ethanol Extraction

The effect of ethanol temperature on the extraction efficiency for cannabis was determined by varying the temperature of the cold ethanol (−20 °C, −40 °C, and room temperature) during extraction. To emulate the industrial reflux cold ethanol extraction (CEE) system, 40 mL ethanol in 50-mL Falcon tubes were stored at either −20 °C and −40 °C for 24 h. Required cryo-ground cannabis biomass to achieve desired sample-to-solvent ratios were added to the cold ethanol and placed on a Corning LSE variable speed vortex mixer (Corning, Glendale, AZ, USA). Cold ethanol extraction was done by placing the vortex mixer with the sample soaked in ethanol in a freezer at the required temperature. Extractions were carried out with different sample-to-solvent ratios, extraction temperatures, and extraction times. The sample-to-solvent ratios used for this study were calculated by varying cannabis biomass (g) within 40 mL of ethanol with Equation (1).

(1) $$\mathrm{Cannabis}\;\mathrm{biomass}\;\mathrm{in}\;\mathrm{grams}=40\;\mathrm{mL}\;\times \frac{\mathrm{Density}\;\mathrm{of}\;\mathrm{ethanol}\;\left(0.789\frac{g}{\mathrm{mL}}\right)}{\mathrm{mass}\;\mathrm{of}\;\mathrm{ethanol}\;\left(g\right)}\;$$

### 3.4. Calculation of Extraction Yield and Efficiency

After extraction, each extract containing the solvent and cannabis biomass mixture was subjected to vacuum filtration using Whatman 4 filter paper (Sigma Aldrich, St. Louis, MO, USA) to remove any residual biomass. Vacuum rotary evaporator operating at 35 rpm and 50 °C was used to evaporate the ethanol present in the extract to determine the yield of crude cannabis oil. Extraction yield of the crude cannabis oil was calculated using Equation (2). Extraction efficiency at the optimal condition was calculated based on THCA concentration using Equation (3).

(2) $$\mathrm{Yield}\;\left(g\;100{\;g\;\mathrm{dry}\;\mathrm{matter}}^{-1}\right)=\frac{\mathrm{mass}\;\mathrm{of}\;\mathrm{extracted}\;\mathrm{crude}\;\mathrm{cannabis}\;\mathrm{oil}\;\left(g\right)}{\mathrm{mass}\;\mathrm{of}\;\mathrm{dried}\;\mathrm{sample}\;\left(100\;g\right)}\;$$

(3) $$\mathrm{Efficiency}\;\left(\%\right)=\frac{\mathrm{Concentration}\;\mathrm{of}\;\mathrm{THCA}\;\mathrm{in}\;\mathrm{extract}\;\left(\frac{g}{100\;g\;\mathrm{dry}\;\mathrm{matter}}\right)}{\mathrm{Concentration}\;\mathrm{of}\;\mathrm{THCA}\;\mathrm{in}\;\mathrm{cryo}-\mathrm{ground}\;\mathrm{sample}\;\left(\frac{g}{100\;g\;\mathrm{dry}\;\mathrm{matter}}\right)}\times 100\%\;$$

### 3.5. Cannabinoid Analyses by Liquid Chromatography-Tandem Mass Spectrometer (LC-MS/MS)

A cannabinoid analysis method developed and described previously by the National Research Council of Canada was modified and used for this study [39,40]. Extracted crude cannabis oil samples were centrifuged at 489 relative centrifugal force for 5 min. An aliquot of the supernatant was diluted in methanol based on the initial sample biomass (Table 5) used for the extraction (this sample is referred to as the diluted cannabis extract). Samples, standards, and quality control (QC) samples (100 μL) were transferred to high-pressure liquid chromatography (HPLC) vials containing glass inserts. The internal standard (50 μL, 500 ng mL^−1^ in methanol) was added prior to injection onto the liquid chromatography tandem mass spectrometer (LC-MS/MS) system. The LC-MS/MS system consisted of a HPLC (Ultimate3000; Thermo Fisher Scientific, Waltham, MA, USA) coupled to a triple quadrupole mass spectrometer (TSQ Quantiva; Thermo Fisher Scientific, MA, USA). Chromatographic separation was carried out on C_18_ bonded phase column (Accucore C_18_, 150 mm × 2.1 mm i.d. with 2.6 μm particle size; Thermo Fisher Scientific, MA, USA) maintained at 40 °C and the mobile phases consisted of water/formic acid and acetonitrile/formic acid both mixed in a 1000:1 volume ratio. An injection volume of 1 μL was used for the study.

The MS/MS detection of cannabinoids was performed via electrospray ionization in positive ion mode using quasi-molecular ion to product ion transitions [39]. The LC-MS/MS method includes both acidic and neutral forms of the cannabinoids. The neutral forms ionize only in positive mode while the acidic forms ionize equally well in both positive and negative mode. Using positive ionization mode for both neutral and acidic cannabinoids produced more consistent and more similar signal responses for all cannabinoids and resulted in a simplified method, relative to a polarity-switching method. External calibration standard solutions containing 20 cannabinoids were prepared in methanol at concentrations of 10, 20, 100, 1000, 6000, 9000 and 10,000 ng mL^−1^ with quality control samples prepared at 30, 1500 and 8 000 ng mL^−1^. Linear regression, weighted 1/x^2^, was used for calibration with peak area ratio of cannabinoid and internal standard as the response variable.

### 3.6. Terpene Analysis by Gas Chromatography-Tandem Mass Spectrometer (GC-MS/MS)

For terpene analysis, extracted crude cannabis oil samples were centrifuged at 489 relative centrifugal force for 5 min. An aliquot of the supernatant was diluted in hexane based on the initial sample biomass (Table 5) used for the extraction (referred to as the diluted cannabis extract). Samples, standards, and QC samples (150 μL) were transferred to HPLC vials containing glass inserts and the internal standard (50 μL, 1 μg mL^−1^ of linalool-d_3_ in hexane) was added before injection onto the gas chromatography-tandem mass spectrometer (GC-MS/MS) system (Trace 1310 GC coupled to a TSQ 9000 Triple Quadrupole MS/MS; Thermo Fisher Scientific, MA, USA). An injection volume of 1 μL was used for the study.

Chromatographic separation of the analytes was obtained using the TraceGOLD TG-5SilMS column (30 m x 0.25 mm i.d. with 0.25 μm film thickness; Thermo Fisher Scientific, MA, USA) and helium as the carrier gas. The SSL inlet temperature was held at 250 °C with a deactivated splitless quartz wool single taper liner (78.5 mm × 4 mm i.d. × 6.3 mm o.d.; Thermo Fisher Scientific, MA, USA). A constant inlet flow of 1.5 mL min^−1^ with a split flow of 15 mL min^−1^ and a split ratio of 10 was used. Selected reaction monitoring (SRM) scan type with electron impact ionization mode was used for the tandem mass spectrometer, while the ion source temperature and MS transfer line temperature were held at 300 °C and 250 °C, respectively. The temperature program for the GC oven can be found in Table 6.

Calibration curves (0.005–2.5 µg mL^−1^) were generated using weighted linear regression (1/x) of the peak area ratios (analyte/internal standard) versus the concentration of the calibration standards. The concentration of individual terpenes in extracts was determined using the appropriate calibration curve for the metabolite using the resulting peak area ratios. Monitored ions, ion transitions, and mass spectrometer voltage parameters are listed in Table 7

### 3.7. Experimental Design

A five-level-by-two-variables central composite rotatable statistical design (CCRD) with uniform precision was used to compare cold ethanol extraction at various temperatures (−20 °C, −40 °C, and room temperature) with respect to total yield of extracted cannabis crude oil, extraction efficiency, and cannabinoid and terpene concentrations. Central composite rotatable design (CCRD) was used for the study because the design consists of five levels and able to test forth-order quadratic models. Like central composite designs, Box–Behnken designs are response surface designs that require three levels for each independent variable and can only fit second-order quadratic models [41]. Central composite designs can be classified into three groups namely, circumscribed (CCC), inscribed (CCI), and face centered (CCF) central composite designs [22]. Classification of central composite designs are based on the position of the axial points. The axial (α) points of the CCC are placed outside the set experimental parameter limits. This allows for the determination of the effect of values beyond or below the chosen levels of factors on the experimental dependent values/responses. Inscribed central composite design is used when it is not possible to leave the limits of the independent variables and gives a poor prediction compared to CCC. The CCI design uses the factor settings as the axial points and creates a factorial or fractional factorial design within those limits [42]. Five levels are required for each independent variable for CCC and CCI and both designs are rotatable. For CCF designs, the axial points are at the center of each face of the factorial space, so α = ± 1. CCF requires three levels.

As shown in Table 8, a total of 13 experimental runs consisting of 4 combinations of factorial values, 4 combinations of axial values, and 5 combinations of central values were generated for the study. Axial points were fixed at a distance (α = 2^k/4^, where k represents the number of variables) from the center to ensure rotatability. Axial combinations additionally allowed for the inclusion of quadratic terms in the response surface model. Replication of central point assures a greater uniformity in the precision of response estimation over the experimental design.

### 3.8. Statistical Analysis

Statistical analyses were performed using JMP software (JMP 4.3 SAS Institute Inc.). Least square multiple regression methodology was used to evaluate the relationship between the independent and dependent variables. Two different multiple regression equations were used to fit the second-order polynomial model based on the experimental data for cold ethanol extraction at various extraction temperatures (−20 °C, −40 °C, and room temperature) and Soxhlet extraction. The first model (model A) was a full model that included all the independent variables, as well as their respective quadratic and interactions terms (Equation (4)). Model B, the second model, was a modification of model A to exclude and control for the extraction time and all interaction and quadratic terms that include the extraction time (Equation (5)).

Yj = β_0_ + β_1_X_1_ + β_2_X_2_ + β_11_X_1_X_1_ + β_22_X_2_X_2_ + β_12_X_1_X_2_ (4)

Yj = β_0_ + β_1_X_1_ + β_11_X_1_X_1_ (5)

where Yj represents the predicted response (dependent variables), model intercept (β_0_), linear terms (β_1_ and β_2_), quadratic terms (β_11_ and β_22_), and interaction term (β_12_), and X_1_ (sample (g) solvent (g)^−1^) and X_2_ (extraction time) are the independent variables.

Analysis of variance (ANOVA) was used to investigate the statistical significance of the regression coefficients by conducting the Fisher’s F-test at a 95% confidence level. The statistical significance of the model was improved through a “backward elimination” process, deleting non-significant dependent terms (*p* > 0.05). The correlation coefficient (R^2^) was used to estimate the quality of fit of each model to the responses. Adjusted R^2^ was used to determine the significance of the improved models by estimating the significance of the deleted non-significant dependent terms to the full models. Response surface plot was obtained using the fitted model. The optimal conditions for cold ethanol for the dependent variables were obtained based on modelling and desirability function. All the results from the dependent variables were investigated with multivariate analysis and principal component analysis (PCA) using JMP software (JMP 4.3 SAS Institute Inc.).

### 3.9. Verification of Model

Three experiments were conducted using the optimal extraction conditions with the highest desirability used to verify the model. The experimental and predicted values were compared to determine the validity of the model.

## 4. Conclusions

Cold ethanol extraction conditions were evaluated to increase the extraction yield and the concentration of cannabinoids and terpenes at different temperatures (−20 °C, −40 °C, and room temperature). CCRD was used to optimize two independent factors namely samples (g)-to-solvent (g) ratio (1:2.93 to 1:17.07) and extraction time (5.86 to 34.14 min). Developed predictive models for all responses yielded predictable and reproducible results, and the verification of the models showed a close agreement between the experimental values and the predicted values with a strong correlation ranging from 0.87 to 0.93. CCRD predicted that a set sample-to-solvent ratio of 1:15 over 10 min at the different extraction temperatures would provide the optimum conditions for the extraction of cannabis oil with maximum desirability ranging between 0.77–0.83%. At these optimized conditions, extraction yields (g 100 g dry matter^−1^) were 18.2%, 19.7%, and 18.5% for −20 °C, −40 °C, and room temperature, respectively, according to the desirability function (0.77 to 0.83%). Compared to the reference ground sample, the THCA concentration changed from 17.9 (g 100 g dry matter^−1^) to 15, 17.5, and 18.3 with an extraction efficiency of 83.6%, 97.7%, 102.1% for −20 °C, −40 °C, and room temperature, respectively at the optimal condition. Total terpene was reduced by 54.1% and 32.2% for extraction at −20 °C and room temperature, respectively, compared to extraction at −40 °C. The scree plot from PCA analyses indicated that the first two principal components account for 95.3% of the total variance (PC1 = 88% and PC2 = 7.3%) although no significant differences in cold ethanol extraction at different temperatures were observed. Further research studies on ethanol recovery using centrifugation, press system, and vacuum filtration must be conducted to help reduce the operational cost for cannabis industries.

## Figures and Tables

**Table 1:** Matrix of the central composite rotatable statistical design (CCRD) and observed responses (Yj) for cold ethanol extraction of cannabis biomass at −20 °C, −40 °C, and room temperature (RT), with different sample-to-solvent ratios and extraction time.

Cold Ethanol Extraction	Independent Variables		Response/Dependent Variables (g 100 g Dry Matter^−1^)									
	Sample Solvent^−1^	Extraction Time	THC	THCA	Total THC	CBG	CBGA	Total CBG	THCVA	CBCA	Total Terpenes	Yield
	(X_1_, g 40 mL^−1^)	(X_2_, min)	Y_1_	Y_2_	Y_3_	Y_4_	Y_5_	Y_6_	Y_7_	Y_8_	Y_9_	Y_10_
−20 °C	10.77	20	0.09	3.95	3.55	0.02	0.05	0.06	0.18	0.07	0.32	4.92
−40 °C			0.22	10.07	9.05	0.05	0.14	0.17	0.52	0.18	0.36	8.56
RT			0.31	9.56	8.69	0.05	0.14	0.17	0.48	0.19	0.22	9.84
−20 °C	6.31	10	0.34	13.79	12.44	0.07	0.19	0.24	0.72	0.27	0.22	10.92
−40 °C			0.33	13.27	11.97	0.06	0.19	0.23	0.69	0.25	0.93	15.85
RT			0.28	9.68	8.77	0.05	0.14	0.17	0.49	0.19	0.05	13.79
−20 °C	6.31	30	0.19	7.01	6.34	0.03	0.10	0.12	0.35	0.13	0.77	10.92
−40 °C			0.32	12.71	11.46	0.06	0.19	0.23	0.66	0.24	1.13	15.69
RT			0.27	9.37	8.49	0.05	0.13	0.16	0.46	0.19	0.67	11.71
−20 °C	3.16	5.86	0.32	12.96	11.68	0.06	0.19	0.22	0.67	0.24	0.06	16.46
−40 °C			0.32	12.05	10.89	0.06	0.17	0.21	0.63	0.23	1.13	16.77
RT			0.47	15.32	13.91	0.07	0.23	0.28	0.80	0.30	1.06	17.72
−20 °C	3.16	20	0.29	11.16	10.08	0.05	0.16	0.19	0.58	0.21	0.99	16.77
−40 °C			0.27	12.58	11.31	0.06	0.18	0.22	0.65	0.24	1.18	16.72
RT			0.36	11.64	10.57	0.06	0.17	0.21	0.60	0.22	1.10	17.78
−20 °C	3.16	20	0.35	12.71	11.49	0.06	0.19	0.22	0.66	0.25	1.19	17.41
−40 °C			0.41	18.15	16.32	0.09	0.26	0.32	0.94	0.35	1.20	17.72
RT			0.33	12.36	11.17	0.06	0.18	0.22	0.64	0.25	1.02	17.41
−20 °C	3.16	20	0.41	13.44	12.20	0.07	0.19	0.24	0.71	0.26	1.09	17.09
−40 °C			0.31	13.74	12.36	0.06	0.20	0.24	0.72	0.26	1.17	17.72
RT			0.33	11.97	10.83	0.06	0.17	0.21	0.63	0.24	0.92	18.30
−20 °C	3.16	20	0.27	10.59	9.55	0.05	0.15	0.18	0.54	0.20	1.08	17.03
−40 °C			0.39	15.59	14.05	0.08	0.23	0.28	0.83	0.30	1.11	17.46
RT			0.33	11.50	10.41	0.06	0.17	0.20	0.60	0.23	0.94	17.35
−20 °C	3.16	20	0.26	10.55	9.51	0.05	0.15	0.18	0.53	0.19	1.14	17.41
−40 °C			0.30	11.66	10.53	0.06	0.17	0.21	0.61	0.22	1.14	17.72
RT			0.40	13.24	12.01	0.07	0.19	0.24	0.71	0.26	1.13	17.98
−20 °C	3.16	34.14	0.31	12.80	11.54	0.06	0.18	0.22	0.65	0.23	1.05	16.51
−40 °C			0.33	12.25	11.07	0.06	0.17	0.21	0.63	0.24	1.09	17.09
RT			0.35	11.50	10.43	0.06	0.16	0.20	0.59	0.22	1.09	16.46
−20 °C	2.1	10	0.43	15.65	14.16	0.07	0.23	0.27	0.84	0.31	1.13	17.54
−40 °C			0.50	19.14	17.28	0.09	0.28	0.34	1.02	0.37	2.00	19.62
RT			0.45	17.03	15.38	0.09	0.25	0.31	0.93	0.34	1.27	18.10
−20 °C	2.1	30	0.35	12.70	11.49	0.06	0.18	0.22	0.68	0.26	1.26	18.57
−40 °C			0.41	15.94	14.40	0.07	0.23	0.28	0.82	0.30	2.06	18.45
RT			0.44	15.65	14.16	0.08	0.24	0.29	0.82	0.31	1.16	19.05
−20 °C	1.85	20	0.36	15.20	13.70	0.07	0.22	0.26	0.80	0.28	1.14	18.82
−40 °C			0.45	16.99	15.35	0.08	0.25	0.30	0.92	0.32	2.32	20.11
RT			0.61	20.27	18.39	0.10	0.30	0.37	1.08	0.40	1.23	19.46

**Table 2:** Regression equation coefficients for cold ethanol extraction of cannabis with different experimental conditions.

Response/Dependent Variables		Regression Model Effect Parameters					
		Intercept	Linear		Quadratic		Interaction
		β_0_	β_1_	β_2_	β_11_	β_22_	β_12_
Cold ethanol extraction at −20 °C							
THC	Coefficient	0.32	0.08	−0.03	−0.03	0.01	0.02
	*p* value	<0.0001 *	0.01 *	0.23	0.25	0.6	0.61
THCA	Coefficient	11.69	2.93	−1.24	−0.79	0.86	0.96
	*p* value	<0.0001 *	0.01 *	0.13	0.34	0.3	0.38
Total THC	Coefficient	10.57	2.65	−1.12	−0.72	0.77	0.86
	*p* value	<0.0001 *	0.01 *	0.13	0.34	0.31	0.39
CBG	Coefficient	0.06	0.01	−0.01	−0.01	0	0.01
	*p* value	<0.0001 *	0.02 *	0.16	0.35	0.47	0.23
CBGA	Coefficient	0.17	0.05	−0.02	−0.01	0.01	0.01
	*p* value	<0.0001 *	0.01 *	0.1	0.29	0.3	0.51
Total CBG	Coefficient	0.2	0.05	−0.02	−0.02	0.01	0.02
	*p* value	<0.0001 *	0.01 *	0.16	0.33	0.35	0.39
THCVA	Coefficient	0.6	0.17	−0.07	−0.04	0.05	0.05
	*p* value	<0.0001 *	0 *	0.12	0.39	0.32	0.38
CBCA	Coefficient	0.22	0.06	−0.03	−0.01	0.02	0.02
	*p* value	<0.0001 *	0.01 *	0.13	0.41	0.36	0.32
Total terpenes	Coefficient	1.1	0.32	0.26	−0.13	−0.22	−0.11
	*p* value	<0.0001 *	0.001 *	0.002 *	0.06	0.01 *	0.23
Extraction yield	Coefficient	17.14	4.24	0.14	−2.56	−0.25	0.26
	*p* value	<0.0001 *	<0.0001 *	0.63	<0.0001 *	0.42	0.53
Cold ethanol extraction at −40 °C							
THC	Coefficient	0.34	0.07	−0.01	0.01	0.01	−0.02
	*p* value	<0.0001 *	0.01 *	0.62	0.54	0.68	0.52
THCA	Coefficient	14.34	2.36	−0.43	0.2	−0.49	−0.66
	*p* value	<0.0001 *	0.03 *	0.63	0.83	0.61	0.60
Total THC	Coefficient	12.91	2.14	−0.39	0.19	−0.42	−0.59
	*p* value	<0.0001 *	0.03 *	0.62	0.82	0.62	0.6
CBG	Coefficient	0.07	0.01	0	0	0	−0.01
	*p* value	<0.0001 *	0.04 *	0.57	0.89	0.51	0.42
CBGA	Coefficient	0.21	0.04	−0.01	0	−0.01	−0.01
	*p* value	<0.0001 *	0.03 *	0.64	0.81	0.53	0.51
Total CBG	Coefficient	0.25	0.04	−0.01	0	−0.01	−0.01
	*p* value	<0.0001 *	0.03 *	0.64	0.89	0.56	0.51
THCVA	Coefficient	0.75	0.13	−0.03	0.02	−0.03	−0.04
	*p* value	<0.0001 *	0.02 *	0.54	0.75	0.56	0.52
CBCA	Coefficient	0.27	0.05	−0.01	0	−0.01	−0.02
	*p* value	<0.0001 *	0.03 *	0.65	0.99	0.69	0.55
Total terpenes	Coefficient	1.16	0.6	0.03	0.17	0.05	−0.04
	*p* value	<0.0001 *	<0.0001 *	0.73	0.06	0.52	0.73
Extraction yield	Coefficient	17.47	2.87	−0.09	−1.12	0.18	−0.22
	*p* value	<0.0001 *	0.01 *	0.88	0.12	0.78	0.8
Cold ethanol extraction at room temperature							
THC	Coefficient	0.35	0.1	0.02	0.04	0.01	0
	*p* value	<0.0001 *	0.01 *	0.24	0.11	0.58	1
THCA	Coefficient	12.14	3.6	−0.9	1.08	0.33	−0.27
	*p* value	<0.0001 *	<0.0001 *	0.04 *	0.02 *	0.42	0.61
Total THC	Coefficient	11	3.25	−0.8	0.98	0.3	−0.24
	*p* value	<0.0001 *	<0.0001 *	0.04 *	0.03 *	0.4	0.63
CBG	Coefficient	0.06	0.02	0	0.01	0	0
	*p* value	<0.0001 *	<0.0001 *	0.05	0.01 *	0.55	0.22
CBGA	Coefficient	0.18	0.06	−0.01	0.02	0.01	0
	*p* value	<0.0001 *	<0.0001 *	0.03 *	0.02 *	0.42	1
Total CBG	Coefficient	0.22	0.07	−0.02	0.02	0.01	0
	*p* value	<0.0001 *	<0.0001 *	0.04 *	0.03 *	0.43	0.81
THCVA	Coefficient	0.64	0.21	−0.05	0.06	0.01	−0.02
	*p* value	<0.0001 *	<0.0001 *	0.02 *	0.03 *	0.51	0.47
CBCA	Coefficient	0.24	0.07	−0.02	0.02	0.01	−0.01
	*p* value	<0.0001 *	<0.0001 *	0.04 *	0.02 *	0.53	0.47
Total terpenes	Coefficient	1.02	0.39	0.07	−0.18	0	−0.18
	*p* value	<0.0001 *	<0.0001 *	0.14	0.01 *	0.97	0.02 *
Extraction yield	Coefficient	17.76	3.16	−0.36	−1.61	−0.39	0.76
	*p* value	<0.0001 *	<0.0001 *	0.04 *	<0.0001 *	0.04 *	0.01 *

Effects are statistically significant if *p* value * < 0.05. Model intercept (β_0_), linear terms (β_1_ and β_2_), quadratic terms (β_11_ and β_22_), and interaction term (β_12_) are the model effect parameters.

**Table 3:** Optimal experimental conditions for cold ethanol extraction of cannabis at −20 °C, −40 °C, and room temperature and the predicted response values.

Extraction Method	Cold Ethanol Extraction at −20 °C	Cold Ethanol Extraction at −40 °C	Cold Ethanol Extraction at Room Temperature
Desirability	0.83	0.77	0.78
Optimal independent experimental conditions			
Sample (g)-to-solvent (40 mL)	2.1	2.1	2.1
Sample (g)-to-solvent (g)	1/15	1/15	1/15
Extraction time (min)	10	10	10
Predicted response values at optimal conditions (g 100 g dry matter^−1^)			
THC	0.39	0.46	0.52
THCA	14.98	17.51	18.30
Total THC	13.53	15.81	16.56
CBG	0.07	0.08	0.09
CBGA	0.22	0.25	0.27
Total CBG	0.26	0.31	0.33
THCVA	0.79	0.93	0.99
CBCA	0.29	0.34	0.36
Total terpenes	0.91	1.98	1.34
Extraction yield	18.18	19.72	18.53
Extraction efficiency (%)	83.61	97.73	102.14

**Table 4:** Analysis of variance (ANOVA) analyses of responses for cold ethanol extraction at different temperatures.

Response (g 100 g Dry Matter^−1^)	Source						F Ratio	Prob > F	Lack-of-Fit (Prob > F)	R^2^	Adjusted R^2^
	Model			Residual							
	df	SS	MS	df	SS	MS					
Cold ethanol extraction at −20 °C											
THC	5	0.07	0.01	7	0.03	0.00	3.1	0.09 (0.01 *)	0.41 (0.31)	0.69	0.47
THCA	5	95.79	19.16	7	29.17	4.17	4.6	0.04 *	0.1	0.77	0.6
Total THC	5	78.22	15.64	7	24.05	3.44	4.55	0.04 *	0.1	0.76	0.6
CBG	5	0.002	0.0004	7	0.001	0.0001	3.2	0.08 (0.03 *)	0.21 (0.4)	0.7	0.48
CBGA	5	0.02	0.004	7	0.01	0.001	5.23	0.03 *	0.14	0.79	0.64
Total CBG	5	0.03	0.01	7	0.01	0.001	3.99	0.04 *	0.13	0.74	0.56
THCVA	5	0.3	0.06	7	0.09	0.01	4.69	0.03 *	0.13	0.77	0.61
CBCA	5	0.04	0.01	7	0.01	0.002	4.24	0.04 *	0.16	0.75	0.57
Total terpenes	5	1.82	0.36	7	0.17	0.02	14.46	0.001 * (0.03 *)	0.03 * (0.98)	0.91	0.85
Extraction yield	5	189.89	37.98	7	4.23	0.6	62.81	<0.001 *	0.01 *	0.98	0.96
Cold ethanol extraction at −40 °C											
THC	5	0.05	0.01	7	0.02	0.00	2.74	0.11 (0.01 *)	0.54 (0.24)	0.66	0.42
THCA	5	50.01	10	7	40.58	5.80	1.73	0.25 (0.03 *)	0.6 (0.53)	0.55	0.23
Total THC	5	41.09	8.22	7	32.74	4.68	1.76	0.24 (0.03 *)	0.6 (0.52)	0.56	0.24
CBG	5	0.001	0.0002	7	0.001	0.0001	1.55	0.29 (0.04 *)	0.84 (0.88)	0.53	0.19
CBGA	5	0.01	0.002	7	0.01	0.001	1.83	0.23 (0.03 *)	0.52 (0.48)	0.57	0.26
Total CBG	5	0.02	0.003	7	0.01	0.002	1.78	0.24 (0.03 *)	0.55 (0.48)	0.56	0.24
THCVA	5	0.16	0.03	7	0.11	0.02	2.05	0.19 (0.03 *)	0.61 (0.59)	0.59	0.31
CBCA	5	0.02	0.004	7	0.02	0.002	1.68	0.26 (0.03 *)	0.6 (0.52)	0.55	0.22
Total terpenes	5	3.05	0.61	7	0.28	0.04	15.38	0.001 *	<0.001 * (0.21)	0.92	0.86
Extraction yield	5	75.73	15.15	7	19.30	2.76	5.49	0.02 *	0.01 *	0.80	0.65
Cold ethanol extraction at room temperature											
THC	5	0.09	0.02	7	0.03	0	6.5	0.01 *	0.96	0.82	0.7
THCA	5	118.43	23.69	7	6.98	1	23.74	<0.001 *	0.13	0.94	0.9
Total THC	5	96.78	19.36	7	6	0.86	22.58	<0.001 *	0.12	0.94	0.9
CBG	5	0.003	0.001	7	0.0001	0.00001	41.58	<0.001 *	0.86	0.97	0.94
CBGA	5	0.03	0.01	7	0.002	0.0002	23.54	<0.001 *	0.06	0.94	0.9
Total CBG	5	0.04	0.01	7	0.003	0.0004	21.9	<0.001 *	0.18	0.94	0.9
THCVA	5	0.39	0.08	7	0.02	0.003	28.1	<0.001 *	0.28	0.95	0.92
CBCA	5	0.05	0.01	7	0.003	0.0004	23.64	<0.001 *	0.22	0.94	0.9
Total terpenes	5	1.62	0.32	7	0.1	0.01	23.32	<0.001 *	0.21	0.94	0.90
Extraction yield	5	101.31	20.26	7	1.25	0.18	113.67	<0.001 *	0.39	0.99	0.98

* Statistically significant (*p* < 0.05). *p*-values for ANOVA, and Lack-of-fit for the revised model, model B, which excludes the extraction time and all interaction and quadratic terms that include the extraction time are shown in parenthesis. Degree of freedom (df), Sum of squares (SS), and Mean square (MS).

**Table 5:** Dilution factors used for cannabinoid and terpene analyses for the extracted sample biomass.

Dilution Factor	Approximate Initial Mass of Biomass (g)
Cannabinoid analysis	
5000-fold	10
3000-fold	6
1500-fold	3
1000-fold	2
Terpene analysis	
1000-fold	10
500-fold	6
200-fold	3
100-fold	2

**Table 6:** Gas chromatography oven temperature program.

Retention Time (min)	Rate (°C min^−1^)	Target Value (°C)	Hold Time (min)
2.000	0.00	65.0	2.00
8.000	10.00	125.0	0.00
18.333	15.00	250.0	2.00
25.000	30.00	300.0	5.00
25.000	Stop Time		

**Table 7:** Gas chromatography-tandem mass spectrometer acquisition parameters for terpenes.

Name	Q1 (*m*/*z*)	Q3 (*m*/*z*)	CE (eV)	Q1 (*m*/*z*)	Q3 (*m*/*z*)	CE (eV)	RT (min)
α-pinene	*93.1*	*77.1*	*10*	93.1	91.1	6	4.1
camphene	*93.1*	*77.1*	*12*	121.1	93.1	8	4.4
β-pinene	*93.1*	*77.1*	*10*	93.1	91.1	6	4.8
β-myrcene	*93.1*	*77.1*	*10*	93.1	91.1	6	4.9
Δ-3-carene	*93.1*	*77.1*	*10*	105.1	79.1	7	5.3
α-terpinene	*136.1*	*121.1*	*10*	121.1	93.1	8	5.4
p-isopropyl toluene	*134.1*	*119.1*	*6*	119.1	117.1	8	5.5
d-limonene	*121.1*	*93.1*	*8*	93.1	77.1	12	5.6
eucalyptol	*108.1*	*93.1*	*5*	108.1	77.1	20	5.7
ocimene	*93.1*	*77.1*	*10*	121.1	93.1	5	5.8
γ-terpinene	*136.1*	*121.1*	*7*	136.1	93.1	8	6.1
terpinolene	*136.1*	*121.1*	*8*	136.1	93.1	8	6.5
linalool	*93.1*	*77.1*	*10*	93.1	91.1	5	6.6
isopulegol	*121.1*	*93.1*	*8*	111.1	55.1	10	7.5
geraniol	*69.1*	*41.0*	*5*	69.1	39.0	14	8.9
β-caryophyllene	*133.1*	*91.1*	*8*	133.1	105.1	8	10.9
α-humulene	*93.1*	*77.1*	*10*	93.1	91.1	6	11.3
nerolidol 1	*136.1*	*121.1*	*5*	93.1	77.1	12	11.9
nerolidol 2	*136.1*	*121.1*	*5*	93.1	77.1	12	12.12
caryophyllene oxide	*93.1*	*91.1*	*8*	121.1	93.1	5	12.5
guaiol	*161.1*	*105.1*	*8*	161.1	119.1	8	12.5
α-bisabolol	*109.1*	*67.1*	*7*	119.1	91.1	12	13.2
linalool-d_3_	*74.07*	*43.1*	*8*	96.1	79.1	10	6.6

Italic values indicate quantitation ion parameters and non-italic values indicate confirmation ion parameters. Q1 (*m*/*z*) and Q3 (*m*/*z*) are the mass-to-charge ratios of the molecular ion selected in Q1 and the fragment ion selected in Q3, respectively. CE is the collision energy and RT is the chromatographic retention time of each terpene.

**Table 8:** Rotatable central composite design in the coded ^A^ and uncoded form of the independent variables (X_1_ and X_2_) for cold ethanol extraction at different temperatures (−20 °C, −40 °C, and room temperature).

Run	Sample (g) Solvent (g)^−1^ (*w*/*w*)	Sample (g) Solvent (40 mL)^−1^ (*w*/*v*) (X_1_)	Extraction Time (min) (X_2_)
1	1/2.93	10.77 (−1.414)	20 (0)
2	1/5	6.31 (−1)	10 (−1)
3	1/5	6.31 (−1)	30 (+1)
4	1/10	3.16 (0)	5.86 (−1.414)
5	1/10	3.16 (0)	20 (0)
6	1/10	3.16 (0)	20 (0)
7	1/10	3.16 (0)	20 (0)
8	1/10	3.16 (0)	20 (0)
9	1/10	3.16 (0)	20 (0)
10	1/10	3.16 (0)	34.14 (+1.414)
11	1/15	2.1 (+1)	10 (−1)
12	1/15	2.1 (+1)	30 (+1)
13	1/17.07	1.85 (+1.414)	20 (0)

^A^ Values in parentheses represent coded forms of the variables.

## Data Availability

Not applicable.
